# FastUniq: A Fast *De Novo* Duplicates Removal Tool for Paired Short Reads

**DOI:** 10.1371/journal.pone.0052249

**Published:** 2012-12-20

**Authors:** Haibin Xu, Xiang Luo, Jun Qian, Xiaohui Pang, Jingyuan Song, Guangrui Qian, Jinhui Chen, Shilin Chen

**Affiliations:** 1 The National Engineering Laboratory for Breeding of Endangered Medicinal Materials, Institute of Medicinal Plant Development, Chinese Academy of Medical Sciences & Peking Union Medical College, Beijing, People’s Republic of China; 2 Key Laboratory of Forest Genetics and Biotechnology, Ministry of Education of China, Nanjing Forestry University, Nanjing, Jiangsu Province, China; 3 Department of Geosciences, Stony Brook University, Stony Brook, New York, United States of America; Natural Resources Canada, Canada

## Abstract

The presence of duplicates introduced by PCR amplification is a major issue in paired short reads from next-generation sequencing platforms. These duplicates might have a serious impact on research applications, such as scaffolding in whole-genome sequencing and discovering large-scale genome variations, and are usually removed. We present FastUniq as a fast *de novo* tool for removal of duplicates in paired short reads. FastUniq identifies duplicates by comparing sequences between read pairs and does not require complete genome sequences as prerequisites. FastUniq is capable of simultaneously handling reads with different lengths and results in highly efficient running time, which increases linearly at an average speed of 87 million reads per 10 minutes. FastUniq is freely available at http://sourceforge.net/projects/fastuniq/.

## Introduction

Massively parallel sequencing technologies, also called next-generation sequencing (NGS) technologies, provide a major approach to obtaining millions of short reads from DNA/RNA samples. NGS has been used in a wide range of research areas over the past few years such as determining whole-genome sequences for new species [1], [2], addressing evolutionary processes at a genomic scale in natural populations [3], identifying mutant alleles in oncogenes in human cancers [4], and resolving whole-genome transcription profiles [5].

In general, the quality of NGS data is one of the major concerns with final study conclusions. Thus, quality control is generally considered the first step in data analyses and is a mandatory prerequisite to downstream analyses and further studies [6]. The presence of duplicates is a major issue in paired short reads from NGS platforms. Polymerase chain reaction (PCR) amplification is one of the major sources of duplicates, which are usually introduced during sequencing library amplification [7]. These duplicates might have a serious impact on research applications, such as scaffolding in whole-genome sequencing [2] and discovering large-scale genome variations [8], and are usually removed. For example, scaffolding is one of the key steps in whole-genome sequencing, in which paired read mappings are used to estimate the order and intervening distance between initial contiguous sequences (contigs) [9]. Because the number of read pairs spanning contigs plays critical roles in scaffolding results, two types of errors may be introduced by the existence of duplicates: false-positive results, in which contigs are incorrectly connected due to the increased numbers of connections; and false-negative results, in which contigs are incorrectly unconnected due to the increased numbers of conflicting connections.

In recent studies, pipelines using a mapping-based strategy have been used to remove duplicates in paired short reads [2], [10]–[14]. In this process, read pairs are first aligned to reference sequences using short read alignment tools such as Bowtie [15], Crossbow [16], and BWA [17], and those read pairs that are exactly mapped to the same position are considered duplicate candidates. Duplicates of this kind are finally removed using tools such as Rmdup in the SAMtools package [18], MarkDuplicates in the Picard toolkit [19], and SEAL [20].

In many studies, however, the performance of the mapping-based strategy is not always satisfactory. In most cases, mapping-based strategies require completed genome sequences as references, and thus, they are not suitable for the many species without genome sequences available. More importantly, the accuracy of paired reads alignment might be affected both by genomic variations that are widely distributed among individuals such as large scale structural variations [21], copy number variations [22], small insertion/deletion variations [23], and single-nucleotide polymorphisms (SNPs) [3], and by repetitive elements that are interspersed throughout the genome such as *Alu* elements in primate genomes [24] and *Mu* transposons in plant genomes [25]. Hence, a mapping-based strategy is not sufficient in many studies of model species and especially in studies focusing on genomic variations and genomes containing large numbers of repeat elements. Thus, a tool is required that implements a *de novo* strategy to remove duplicates only by making use of clues in paired short reads from NGS platforms, regardless of the availability of completed genome sequences. Recently, a *de novo* strategy was implemented in several tools such as fastx_collapser in the FASTX-Toolkit [26] and Fulcrum [27] was used successfully to remove duplicates. However, these tools are either not designed for the removal of duplicates in paired short reads, or inefficient with running time ranged from several hours to several days.

To accelerate duplicates removal in paired short reads using a *de novo* strategy, we developed FastUniq. FastUniq is a fast tool that can handle data at an average speed of 87 million reads per 10 minutes.

## Program Design

**Figure 1:**
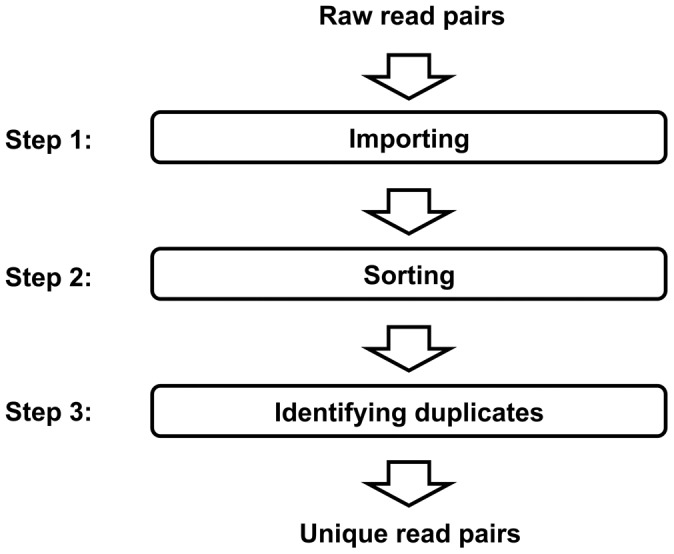
The processing flow chart for FastUniq. Step 1: import all read pairs into memory; Step 2: sort read pairs based on nucleotide sequences; Step 3: identify duplicates in sorted read pairs and output the unique sequences.

FastUniq was engineered to accomplish duplicate read-pair removal in a three-step process (Figure 1). First, FastUniq imports all paired reads into memory. Then, FastUniq sorts these read pairs on the basis of their sequences. Finally, FastUniq marks duplicates in sorted read pairs and outputs the unique sequences.

FastUniq accepts a list of FASTQ sequence files as its input file, in which two adjacent files with reads in the same order belong to a pair. FastUniq can simultaneously handle reads with different lengths. FastUniq outputs unique read pairs into two sequence files in either FASTQ or FASTA format, with reads in the same order belonging to a pair. In addition, FastUniq provides an option to output unique read pairs into a single sequence file in FASTA format with adjacent sequences belonging to a pair.

FastUniq was written in C language using standard POSIX libraries and can be run at full speed on most UNIX/Linux-compatible systems.

## Implementation

### Importing Paired Short Reads

**Figure 2:**
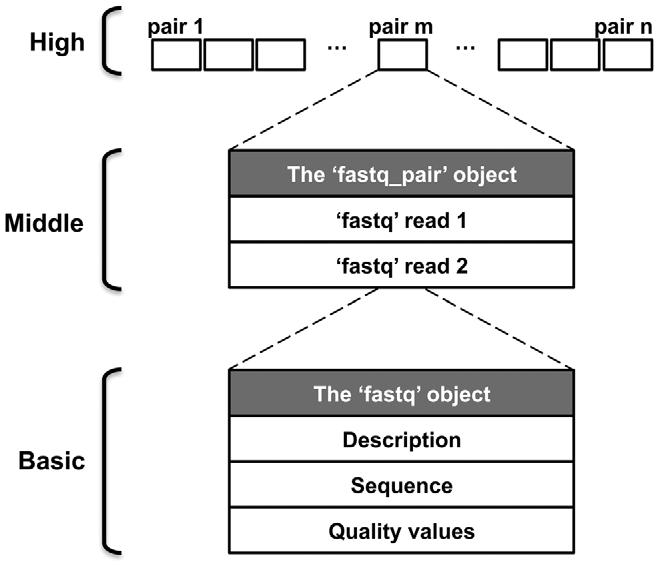
FastUniq three-tier architecture for storage of read pairs. The high-tier objective was to store hundreds of millions or more of paired reads. Data for each read pair composed of two reads are stored in a middle-tier ‘fastq_pair’ object, and data for each read are stored in a basic-tier ‘fastq’ object.

FastUniq imports all read pairs into memory. In this process, a three-tier architecture was built to store hundreds of millions or more of paired reads (Figure 2). In the basic tier, the object named ‘fastq’ is used to store data for one read, including description, sequence, and quality values. The ‘fastq_pair’ middle-tier object is composed of two ‘fastq’ objects to store data for a read pair, and the high-tier object is a list composed of large numbers of ‘fastq_pair’ objects. After all paired reads are correctly imported, the list of ‘fastq_pair’ is indexed for rapid access to any ‘fastq_pair’ objects in the list.

### Sorting

FastUniq makes use of the merge sort algorithm [28] to sort all ‘fastq_pair’ objects in the list. The order of ‘fastq_pair’ objects is determined by nucleotide sequences in paired reads. To compare two ‘fastq_pair’ objects, FastUniq first compares sequences of the first reads and then compares sequences of the second reads only if sequences of the first reads were the same. To determine the order of two sequences, FastUniq compares bases at the same position in a one-by-one manner using the sorting rule of ‘A’, ‘C’, ‘G’, and ‘T’ in order; the order of these two sequences is determined when the first different bases are detected. For two sequences with different lengths, the order is determined by the sequence length if the shorter sequence exactly matches to the 5′ end of the longer one.

### Removing Duplicates and Outputting the Unique Sequences

FastUniq identifies duplicates in the sorted ‘fastq_pair’ list by comparing the adjacent read pairs in the list. Similarly, duplicates also are identified by sequence comparison. Two reads with different lengths are considered the same if the shorter sequence exactly matches to the 5′ end of the longer one, and two read pairs are identified as duplicate candidates if both reads are considered the same. For two duplicate pairs, FastUniq outputs the one in which the lengths of both reads are longer than or equal to another; otherwise, FastUniq outputs both reads.

## Application

We evaluated FastUniq using Illumina sequencing libraries of the *Acropora digitifera* genome project [2], taken from the DDBJ Read Archive (DRA000447) [29] and including all paired-end libraries corresponding to short insert sizes of 200, 300, 500, and 700 base pair (bp) and all mate-pair libraries corresponding to large insert sizes of 1, 3, 5, 7, 15, and 20 kilobase (kb). Nucleotides were trimmed from the end of reads with the fastq_quality_trimmer in the FASTX-Toolkit [26], with a quality threshold of 20 and a length threshold of 20 bp, in a one-by-one manner. The clean read pairs then were extracted.

**Figure 3:**
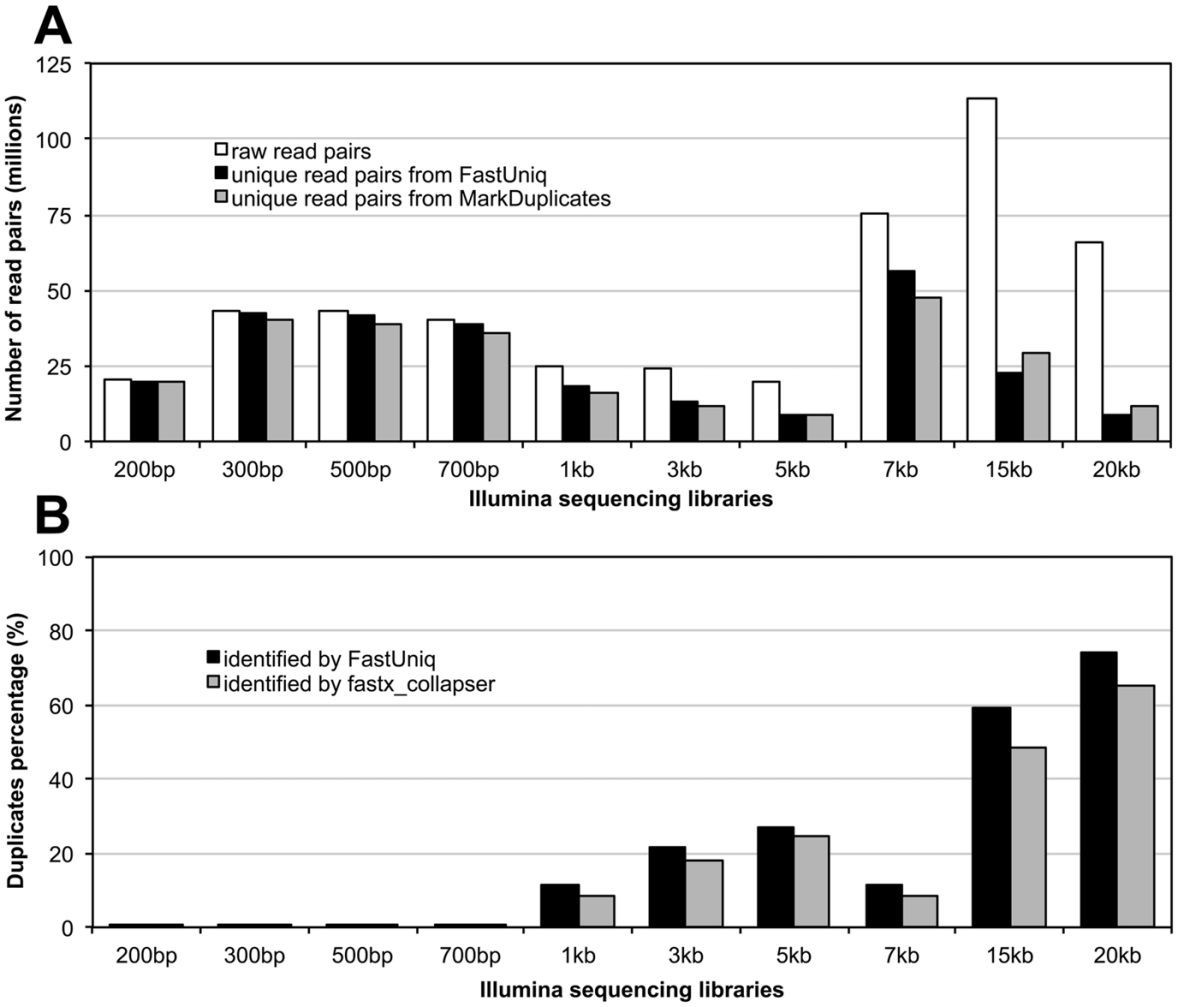
Results of duplicates removal for Illumina sequencing libraries from *Acropora digitifera* corresponding to multiple insert sizes. (A) The number of read pairs before and after duplicates removal using FastUniq or the mapping-based pipeline for each library. (B) The percentage of duplicates in the results of the mapping-based pipeline identified using FastUniq or fastx_collapser for each library.

Duplicates were identified from these paired reads on a DELL PowerEdge R910 server with 256 gigabytes (GB) RAM. The maximum memory usage is 35.6 GB at the time of removing duplicates for mate-pair library corresponding to a 15-kb insert size (DDBJ:DRX000986), which was composed of 227 million reads or 16.6 billion bases. Figure 3A shows the levels of duplicates identified by FastUniq for each library, in which levels of duplicates are obviously different between paired-end libraries and mate-pair libraries (Table S1). Of these, all paired-end libraries had levels of duplicates lower than 4%, indicating the success of preparation steps for these paired-end libraries. In contrast, all mate-pair libraries had significantly higher levels of duplicates, in which the lowest ones were 25% in both the 1-kb and the 7-kb libraries. In particular, the highest levels exceeded 80% in both the 15-kb and the 20-kb libraries.

## Evaluation

### Accuracy

The fastx_collapser in the FASTX-Toolkit is a widely accepted *de novo* tool for removing duplicates from unpaired reads [26]. For libraries composed of paired reads with identical read length, duplicates could also be removed accurately using fastx_collapser through merging reads belonging to a pair into a single sequence.

To evaluate the accuracy of FastUniq, duplicates were identified in the clean read pairs of *Acropora digitifera* mate-pair library corresponding to a 15-kb insert size with identical read length of 75 bp (DDBJ:DRX000986) using both FastUniq and fastx_collapser. By comparing the results, we found the unique read pairs identified by FastUniq was identical to that identified by fastx_collapser (data not shown). The result indicates that FastUniq has a good accuracy on paired short reads.

### Comparison with the Mapping-based Strategy

To verify the conclusions from the application of FastUniq, a mapping-based pipeline consisting of BWA and the Picard toolkit was used to identify duplicates from the same data sets, using the completed genome sequences of *Acropora digitifera* [2], taken from Okinawa Institute of Science and Technology [30], as its references. The details of the procedures are as follows. First, read pairs for each library were aligned to references using a pipeline composed of the ‘index’, ‘aln’, and ‘sampe’ functions of BWA in sequence to generate an index for reference. Next, the suffix array (SA) coordinates of good hits of each individual read were found. Finally, the SA coordinates of paired reads were converted to chromosomal coordinates. The results were stored in sequence alignment/map (SAM) format [18]. Then, duplicates were identified based on their coordinate position relationships stored in SAM files, using a pipeline consisting of the ‘SortSam’, ‘MarkDuplicates’, and ‘SamToFastq’ functions of the Picard toolkit in the sequence to coordinately sort read pairs. Duplicates then were removed from the coordinated sorted pairs, and the unique pairs were exported in FASTQ format.

Because both the Illumina sequencing libraries and the completed genome were from a single clonal colony of the coral *Acropora digitifera* species [2], the negative effects of the Picard MarkDuplicates results were reduced to a minimum level. Thus, Picard MarkDuplicates performs well in removing duplicates from these sequencing libraries. Therefore, the fact that the level of duplicates identified by FastUniq for each library was close to or exceeding that identified by Picard MarkDuplicates (Figure 3A, Table S1) indicates that FastUniq performs well on these sequencing libraries.

To further evaluate the effect of FastUniq, we used this software to check the level of duplicates for each library after mapping-based duplicates were removed. We found that duplicates existed in all libraries after mapping-based duplicates were removed and especially in mate-pair libraries corresponding to large insert sizes of 15 kb and 20 kb, in which the level of duplicates remained 60% and 74%, respectively (Figure 3B, Table S2). Meanwhile, we merged reads belonging to a pair into a single sequence for each library after mapping-based duplicates were removed, and then used fastx_collapser in the FASTX-Toolkit to check the level of duplicates with exactly the same sequence in both reads in pairs for each library. The similar trends of duplicate percentages identified by FastUniq and fastx_collapser (Figure 3B, Table S2) confirmed the existence of duplicates in the results of the mapping-based strategy.

Several reasons may contribute to the existence of these duplicates. To determine the major ones, read pairs not mapped to references in the mate-pair library corresponding to a 15-kb insert size (DDBJ:DRX000986) were extracted after mapping-based duplicates were removed, accounting for 56% of the library. The level of duplicates for these unmapped pairs was identified using FastUniq. We found a 79% level of duplicates within these unmapped pairs, accounting for 74% of duplicates in the results of the mapping-based strategy. Thus, it can be concluded that majority of duplicates in the results of the mapping-based strategy was due to the mapping-based strategy lacks the capability to remove duplicates in read pairs not mapped to references.

### Running Time

**Figure 4:**
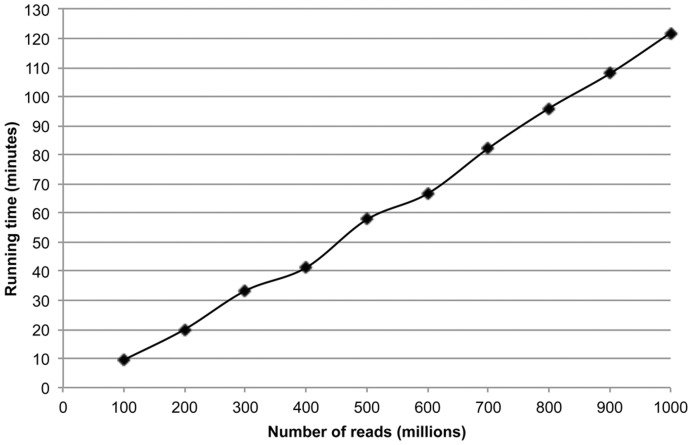
Running time performance of FastUniq. The running time is measured by the ‘time’ command in the Linux operating system.

The running time for FastUniq was evaluated by removing duplicates in a series of libraries, with the number of reads gradually increasing from 100 million to one billion. These libraries were simulated by combining multiple copies of the *Acropora digitifera* mate-pair library corresponding to a 1-kb insert size with read length of 35 bp (DDBJ:DRX000983). The results showed that FastUniq has a highly efficient running time, with the removal of duplicates in a library composed of 100 million reads completed 10 minutes (Figure 4). More importantly, the running time increased linearly with an increasing amount of data, with an average speed of 87 million reads per 10 minutes.

## Discussion

In this paper, we describe a new method that uses a *de novo* strategy to remove duplicates in paired short reads. In recent studies, duplicate removal was generally achieved through pipelines using a mapping-based strategy. Different from the mapping-based strategy in which duplicate candidates were identified based on coordinate relationships between read pairs, the *de novo* strategy determines duplicate candidates directly based on sequences in paired short reads. Thus, the advantage of the *de novo* strategy is that it does not require completed genome sequences as a prerequisite, and it is not influenced by the completion level of genome sequences, widespread genome variation among individuals, or repeat elements in genomes. Therefore, the *de novo* strategy could provide a universal approach to remove duplicates in paired short reads, with a wide adaptability to nearly all species.

We described the implementation of the *de novo* strategy in FastUniq, a tool that can be used with flexibility in almost all NGS-based studies. FastUniq is capable of simultaneously handling reads with different lengths, and thus, it provides an opportunity to remove duplicates in multiple sequencing results from one library and to be integrated into the mainstream NGS processing pipelines. FastUniq can output the unique pairs in multiple sequence formats to meet diverse demands in various types of analyses.

An efficient in-memory architecture was used to store read pairs in FasUniq. In practice, a computing server equipped with 64 GB memory is sufficient for FastUniq to handle reads produced from a whole Illumina Hiseq2000 lane. In general, a 64 GB memory is a minimum requirement for subsequent large-scale data analysis using popular tools such as Velvet [31], SOAPdenovo [32], ALLPATHS-LG [33] and so on. In comparison to the running time of mapping-based pipelines and other *de novo* tools that ranges from several hours to several weeks in our practice, FastUniq is a fast tool that removes duplicates at an average speed of 87 million reads per 10 minutes (Figure 4).

The results from the evaluation demonstrated that FastUniq identified percentages of duplicates close to or exceeding that identified by Picard MarkDuplicates in libraries corresponding to multiple insert sizes from 200 bp to 20 kb (Figure 3A, Table S1). Picard MarkDuplicates performs among the best in this process because of the use of completed genome sequences and minimized genome variations. Theoretically, however, the performance of Picard MarkDuplicates may be significantly reduced in many studies. Because FastUniq only examines bases in paired reads, it can be inferred that FastUniq will show a better performance than Picard MarkDuplicates.

There were some differences in levels of duplicates identified by FastUniq and Picard Markduplicates that were caused by the different criteria in read pair comparisons (Figure 3A, Table 1). Of them, FastUniq compares read pairs on the basis of sequences only, and it is sensitive to SNPs caused by heterozygous or sequencing errors. Picard MarkDuplicates compares read pairs on the basis of coordinate relationships, but in practice, it is not sensitive to a few SNPs between read pairs. In addition, FastUniq identified up to 74% of the levels of duplicates in mate-pair library corresponding to a 20 kb insert size in the Picard MarkDuplicates results (Figure 3B, Table S2), a result that is mainly caused by the inability of the mapping-based strategy to identify duplicates in read pairs that are not mapped to references. Therefore, we conclude that FastUniq is an unbiased tool for removal of duplicates in all input read pairs that maximally retain polymorphisms in the sequencing data.

### Availability

FastUniq is open source software that is freely available at http://sourceforge.net/projects/fastuniq/.

## Supporting Information

Table S1 **The number and percentage of unique read pairs after duplicates removal using FastUniq or the mapping-based pipeline for each library.**
(DOC)

Table S2 **The number and percentage of duplicates in the results of the mapping-based pipeline identified using FastUniq or fastx_collapser for each library.**
(DOC)
