# Supplementary material for: FastUniq: A Fast De Novo Duplicates Removal Tool for Paired Short Reads
Source: PLoS One. 2012 Dec 20;7(12):e52249. doi: 10.1371/journal.pone.0052249 (PMC3527383; doi:10.1371/journal.pone.0052249)
Supplement: Table S1 — The number and percentage of unique read pairs after duplicates removal using FastUniq or the mapping-based pipeline for each library. (DOC) [file pone.0052249.s001.doc]

**Table S1. The number and percentage of unique read pairs after duplicates removal using FastUniq or the mapping-based pipeline for each library.**

| **Libraries** | **Raw read pairs** | **Unique read pairs from FastUniq** | | **Unique read pairs from MarkDuplicates** | |
| --- | --- | --- | --- | --- | --- |
|  |  | **Count** | **Percentage (%)** | **Count** | **Percent (%)** |
| 200bp | 20,258,325 | 20,016,488 | 98.8 | 19,373,444 | 95.6 |
| 300bp | 43,163,307 | 42,117,422 | 97.6 | 40,112,923 | 92.9 |
| 500bp | 42,856,298 | 41,466,270 | 96.8 | 38,903,850 | 90.8 |
| 700bp | 40,145,162 | 38,586,517 | 96.1 | 35,670,077 | 88.9 |
| 1kb | 25,079,829 | 18,611,130 | 74.2 | 16,325,364 | 65.1 |
| 3kb | 23,829,880 | 12,874,466 | 54.0 | 11,490,513 | 48.2 |
| 5kb | 19,713,236 | 8,724,360 | 44.3 | 8,846,410 | 44.9 |
| 7kb | 75,354,466 | 56,013,873 | 74.3 | 47,672,025 | 63.3 |
| 15kb | 113,766,021 | 22,571,484 | 19.8 | 28,995,299 | 25.5 |
| 20kb | 65,894,779 | 8,452,766 | 12.8 | 11,793,460 | 17.9 |
