# Supplementary material for: FastUniq: A Fast De Novo Duplicates Removal Tool for Paired Short Reads
Source: PLoS One. 2012 Dec 20;7(12):e52249. doi: 10.1371/journal.pone.0052249 (PMC3527383; doi:10.1371/journal.pone.0052249)
Supplement: Table S2 — The number and percentage of duplicates in the results of the mapping-based pipeline identified using FastUniq or fastx_collapser for each library. (DOC) [file pone.0052249.s002.doc]

**Table S2. The number and percentage of duplicates in the results of the mapping-based pipeline identified using FastUniq or fastx_collapser for each library.**

| **Libraries** | **Initial read pairs** | **Duplicates identified by FastUniq** | | **Duplicates identified by fastx_collapser** | |
| --- | --- | --- | --- | --- | --- |
|  |  | **Count** | **Percentage (%)** | **Count** | **Percentage (%)** |
| 200bp | 19,373,444 | 42,785 | 0.2 | 37,777 | 0.2 |
| 300bp | 40,112,923 | 120,709 | 0.3 | 100,903 | 0.3 |
| 500bp | 38,903,850 | 109,203 | 0.3 | 92,485 | 0.2 |
| 700bp | 35,670,077 | 86,839 | 0.2 | 70,129 | 0.2 |
| 1kb | 16,325,364 | 1,822,996 | 11.2 | 1,376,072 | 8.4 |
| 3kb | 11,490,513 | 2,487,771 | 21.7 | 2,081,887 | 18.1 |
| 5kb | 8,846,410 | 2,396,440 | 27.1 | 2,164,974 | 24.5 |
| 7kb | 47,672,025 | 5,522,089 | 11.6 | 4,006,044 | 8.4 |
| 15kb | 28,995,299 | 17,250,915 | 59.5 | 14,061,369 | 48.5 |
| 20kb | 11,793,460 | 8,752,755 | 74.2 | 7,707,540 | 65.4 |
